# Study on the Influence of Rotation Axis Misalignment of the Exoskeleton Knee Joint on Human Knee Joint Torque

**DOI:** 10.3390/s26165119

**Published:** 2026-08-12

**Authors:** Changlong Jiang, Xiaorong Guan, Zheng Wang, Dingzhe Li, Long He

**Affiliations:** 1 Department of Mechanical Engineering, Nanjing University of Science and Technology, Nanjing 210094, China; jiangchanglong@njust.edu.cn (C.J.); wangzheng19@njust.edu.cn (Z.W.); lidingzhe@njust.edu.cn (D.L.); 2 Zhiyuan Research Institute, Hangzhou 310000, China; helong_zyy@163.com

**Keywords:** exoskeleton joint misalignment, human knee joint moments, human-exoskeleton coupling simulation, human-exoskeleton interaction forces, assistance efficiency

## Abstract

Misalignment between the rotation axis of a lower-limb exoskeleton knee joint and the human knee joint in the sagittal plane introduces additional human-exoskeleton interaction forces, affecting gait and assistance efficiency. This study proposes an equivalent stiffness-damping scheme that simultaneously accounts for the serial characteristics of both the exoskeleton’s strapping and human soft tissues, and establishes a human-exoskeleton coupling model based on OpenSim that allows for precise adjustment of the misalignment magnitude along the sagittal coordinate system. Simulation results indicate that the effects of misalignment exhibit significant directional dependence: at a misalignment of 0.05 m, the peak increase in extension torque in the positive y-axis direction reached 38.9%, while the peak increase in flexion torque in the negative x-axis direction reached 211.2%. The trends in human-exoskeleton interaction torque and electromyographic signals measured experimentally were consistent with the simulation results. Considering that, at a misalignment of 0.02 m, the maximum difference in torque in the negative x-axis direction was 54.65% and the assist efficiency in the positive x-axis direction dropped to −5.13%, it is recommended that the misalignment of the exoskeleton knee joint be controlled within 0.02 m. This provides quantitative evidence for the structural design and wear calibration of the exoskeleton.

## 1. Introduction

Lower-limb exoskeletons have become an important area of research in wearable robotics. Currently, most lower-limb exoskeleton designs simplify the exoskeleton knee joint into a rotational pair parallel to the human sagittal plane, whereas the human knee joint exhibits coupled rolling and sliding motions between the femoral condyle and the tibial condyle, resulting in a non-fixed instantaneous center of rotation and a J-shaped trajectory during flexion and extension. Li et al. [1] proposed a knee exoskeleton that reproduces the coupled rolling-sliding motion of the human knee joint using a single-stage planetary gearset and three coupled interface gears, thereby enhancing the motion fidelity and drive performance of the knee exoskeleton. Guo et al. [2] proposed an adaptive exoskeleton configuration synthesis method based on screw-linear correlation to address the mismatch issue in human exoskeletons, and analyzed and discussed the mechanical mechanisms of the RRR mechanism in assisting knee flexion and extension and reducing knee load. Cevik et al. [3] proposed a new support design method that incorporates passive degrees of freedom to minimize parasitic forces and improve contact stability; they demonstrated its ability to withstand expected stresses through static and dynamic simulations as well as structural analysis. Although biomimetic knee exoskeletons can effectively simulate the J-shaped trajectory of the human knee’s instantaneous center of rotation through their biomimetic structure, they are affected by individual differences among wearers, the exoskeleton’s own weight, the tightness of the straps, and control system delays. Consequently, in the sagittal plane, the exoskeleton’s knee rotation axis inevitably experiences misalignment with that of the human knee, affecting normal walking gait and the efficiency of exoskeletal assistance.

Most existing research on exoskeletons relies on various dynamic simulation software to conduct human-exoskeleton coupling simulations. Seth et al. [4] introduced a biomechanical simulation software package called OpenSim (Stanford University), which can calculate variables that are difficult to measure experimentally and predict new movements in motion control models. Hirschi et al. [5] proposed a method for performing kinematic optimization early in a specific segment of a series-connected robotic system and demonstrated the effectiveness of the optimization through simulation results. Fernandez-Montoya et al. [6] described the development and validation of an analytical modeling tool for a passive lower-back exoskeleton with adjustable force transmission capabilities; this analytical model allows for rapid evaluation of support force transmission capabilities based on various geometric design parameters. Jun Wei et al. [7] coupled a passive backpack exoskeleton with a musculoskeletal model using OpenSim. Through simulation, they analyzed the torque distribution and metabolic activity of key gait-related muscles during exoskeleton wear, thereby validating the exoskeleton’s effectiveness. Khamar et al. [8] described how to establish a human-exoskeleton coupled model in biomechanical software, perform joint simulations using MATLAB, and conduct experimental validation. Favennec et al. [9] simplified a soft back exoskeleton into massless springs attached to a musculoskeletal model and verified the exoskeleton’s effectiveness in reducing lumbar spine load through human-exoskeleton coupled simulations. Meng et al. [10] conducted simulations by adding three types of massless spring actuators to a musculoskeletal model. They identified a passive energy-storage structure at the ankle joint that maximizes the reduction in metabolic expenditure, proposed a hybrid drive cooperative control strategy, and validated its effectiveness. Bermejo-García et al. [11] modeled the cable anchor point as a welded connection to the musculoskeletal model, with the other end of the cable fixed to the position of the backpack actuator, and used human-exoskeleton coupling simulation to investigate the effects of different anchor point locations on the human thigh. Firouzi et al. [12] simplified the BATEX exoskeleton into a massless two-joint actuator attached to a musculoskeletal model and verified the superiority of the BATEX exoskeleton design through human-exoskeleton coupling simulations. Bermejo-Garcia et al. [13] simplified the hip exoskeleton motor into a massless actuator and used a massless cable driver to simulate cable-driven exoskeleton actuation. These components were added to a human lower-limb musculoskeletal model, and human-exoskeleton coupling simulations concluded that cable-assisted devices outperform traditional motor-driven exoskeletons. Wang et al. [14] used muscle contribution and joint stiffness to determine the location and stiffness of energy storage components in a lower-limb exoskeleton and optimized the design of the lower-limb exoskeleton through human-exoskeleton coupling simulation. Sambhav et al. [15] simplified the upper-limb assistive exoskeleton into massless path actuators attached to a musculoskeletal model of the human right upper limb and added objects of varying masses to the hand of the musculoskeletal model to simulate external loads; they verified the effectiveness of the exoskeleton through human-exoskeleton coupling simulations. Nasiri et al. [16] simplified the upper-limb exoskeleton into two one-degree-of-freedom ideal actuators at the elbow and shoulder joints. They added massed objects to the hand of the musculoskeletal model to simulate external loads and verified the practicality of the upper-limb exoskeleton in weight compensation and mass estimation through human-exoskeleton coupling simulations. Marconi et al. [17] simplified an ankle-foot orthosis into mass points and actuators of varying masses. They optimized the mass and distribution of the orthosis by simulating changes in the size and position of the mass points within a human-exoskeleton coupling model. Green et al. [18] coupled an upper-limb rehabilitation robot model with an arm skeletal model and validated the practicality of this coupled model through simulation and experiments. Wei et al. [19] proposed a novel knee exoskeleton structure, coupled the optimized exoskeleton model with a human lower-limb musculoskeletal model, and validated the exoskeleton’s effectiveness through simulation. Delgado et al. [20] analyzed the linear and angular displacement errors between an upper-limb exoskeleton and a human arm musculoskeletal model to investigate the impact of these errors on the lengths of associated tendons, thereby evaluating and quantifying the human limb’s motion and its interaction with the exoskeleton. Nguyen et al. [21] investigated the effects of devices’ mass and internal dynamics in simulations of human motion assistance. The device actuators were modeled in various ways with differing levels of detail, and their findings demonstrated that the electrodynamics of the motors significantly influence the simulation results. Most of the aforementioned studies on human-exoskeleton coupling models treat the actuators of assistive devices acting on the human body as ideal, massless, generalized torque sources. These torques can result in unrealistically high values and change magnitude instantaneously, which may affect the accuracy of the simulation results. Furthermore, most of the aforementioned studies do not account for misalignment between exoskeleton joints and human joints, nor do they quantitatively analyze the impact of exoskeleton joint misalignment on human joint torques. This study aims to quantify the effect of different degrees of misalignment between the exoskeleton’s knee joint rotation axis and the human knee joint rotation axis on the torque at the human knee joint. Therefore, it is not sufficient to simply model a basic assistive actuator; instead, a physical model of the knee exoskeleton must be integrated into the musculoskeletal model to construct a human-exoskeleton coupling model suitable for this study.

Based on the aforementioned research findings and issues, this study selected a human-exoskeleton coupling model built on OpenSim for simulation analysis; biomechanical research using OpenSim is now well-established. Alijanpour et al. [22] proposed gait phase normalization as part of a comprehensive method for independently analyzing amplitude and temporal variations. This approach, which analyzes phase durations and normalizes data to gait phases, overcomes previous limitations and enables a comprehensive analysis of amplitude and temporal variations in time-series gait data, while also being easily adaptable to other tasks. Roelker et al. [23] compared the effects of four OpenSim models (Gait2392, Lower Limb Model 2010, Whole-Body Model, and Whole-Body Model 2016) on estimates of gait mechanics, muscle forces, and muscle activation, demonstrating that Gait2392 is the optimal model for studying the gait of healthy young adults. Brambilla et al. [24] used musculoskeletal models in OpenSim in combination with gait data to simulate seven load conditions and all possible combinations of two parameters, measuring the effects on lower-limb biomechanics through torque, power, and mechanical work. The results indicated that biomechanics are influenced by both speed and load. The study by Moreira et al. [25] provides a comprehensive dataset that simultaneously records both the raw and processed data from 16 healthy participants walking at seven controlled speeds on a 10-m flat surface. The raw data included 3D joint trajectories from 24 reflective markers, ground reaction forces, force plate torques, centers of pressure, and EMG signals from the tibialis anterior, lateral gastrocnemius, biceps femoris, and vastus lateralis muscles. The study by Bakke et al. [26] demonstrated that in OpenSim, when reconstructing skeletal geometry to calculate joint kinematics, shape modeling is more accurate and reproducible than linear scaling, given the same anatomical landmarks. Di Pietro et al. [27] introduced a tool called the Automatic Scaling Tool, designed to improve and accelerate musculoskeletal simulations based on the General Model in OpenSim. Fox et al. [28] compared OpenSim tools for reducing residuals in human running simulations and determined that the MocoTrack method was the best approach for reducing residuals to near-zero levels, albeit at the cost of longer computation times. Sturdy et al. [29] used a multi-heuristic optimization algorithm to accelerate the residual reduction algorithm in OpenSim by tracking weight decisions to reduce residual errors, thereby minimizing the need for manual adjustments. Kang et al. [30] used the Kelvin-Voigt model to represent viscoelastic behavior and estimated the viscoelastic properties of the human thigh under compression through a pendulum test. Hwang et al. [31] used a joint torque sensor to accurately estimate a user’s muscle force; this sensor incorporates measurements of human dynamic effects-such as inertial, Coriolis, and gravitational torques-as well as torque generated by active muscle force. Experimental results demonstrated that the torque sensor can accurately estimate muscle torque under both relaxed and activated muscle conditions. Chiu et al. [32] developed a lower-limb exoskeleton device that integrates kinematics, vibration analysis, biomechanics, and a machine vision-based motion capture system. This device replicates the physiological structure of the human lower limbs, accurately and automatically tracks the user’s movements, assists with weight-bearing walking, and has been experimentally demonstrated to automatically track human gait.

In modeling human-exoskeleton coupling for wearable exoskeletons, misalignment of the knee joint rotation axis is a key factor affecting assessment accuracy. Most existing studies either assume ideal alignment between the exoskeleton and human joint axes or simplify misalignment to a unidirectional static offset. There is a lack of a modeling method capable of systematically and precisely adjusting the direction and degree of misalignment while simultaneously accounting for the actual mechanical characteristics of human-exoskeleton interaction. Furthermore, simulations of human-exoskeleton interaction forces generally rely on empirically set spring-damping parameters, neglecting the respective contributions of stiffness and damping from the human lower limbs’ soft tissues and the exoskeleton’s strapping structure. This severely limits the model’s ability to predict complex misalignment scenarios. To overcome these methodological limitations, this study makes the following core contributions to misalignment modeling: For the first time, we have established a human-exoskeleton coupled model capable of precisely adjusting the position of the exoskeleton’s knee joint rotation axis. This model allows for the quantitative setting of misalignment magnitudes along four independent axes within the human knee’s sagittal plane coordinate system, thereby enabling parametric simulation of misalignments in different directions and of varying degrees. Additionally, we propose an equivalent spring-damper configuration that simultaneously accounts for the stiffness and damping of both the exoskeleton’s strapping and the human lower limbs’ soft tissues. This fundamentally eliminates reliance on empirical parameters, providing clear physical interpretability for the calculation of human-exoskeleton interaction forces. Based on this misalignment model, we used dynamic simulation for the first time to quantitatively reveal the patterns of how the direction and degree of misalignment affect knee joint moments and the exoskeleton’s assistance efficiency, and thereby defined the acceptable safety range for misalignment during structural design. Further misalignment simulation experiments also validated the accuracy of the simulation results, providing direct methodological support and design boundaries for the tolerance design of exoskeleton joints.

The remainder of this paper is organized as follows: Section 2 introduces the construction and simulation methods for the human-exoskeleton coupling model with misalignment of the exoskeleton’s knee joint rotation axis, as well as the experimental protocol for the exoskeleton’s knee joint rotation axis misalignment. Section 3 describes the results of the human-exoskeleton coupling dynamic simulation and the experimental validation results. Section 4 discusses the implications of the human-exoskeleton coupling dynamic simulation results for exoskeleton structural design and the limitations of this study. Section 5 presents the conclusions of this study.

## 2. Simulation and Experimental Methods

This section primarily covers: the division of the human gait cycle and the definition of the knee joint coordinate system in the sagittal plane; the methodology and simulation steps for constructing a human-exoskeleton coupling model of the exoskeleton’s knee joint rotation axis misalignment; and the experimental protocol for the exoskeleton’s knee joint rotation axis misalignment. The following subsections describe these processes in detail.

### 2.1. Gait Cycle Segmentation and Definition of the Knee Joint Coordinate System

**Figure 1:**
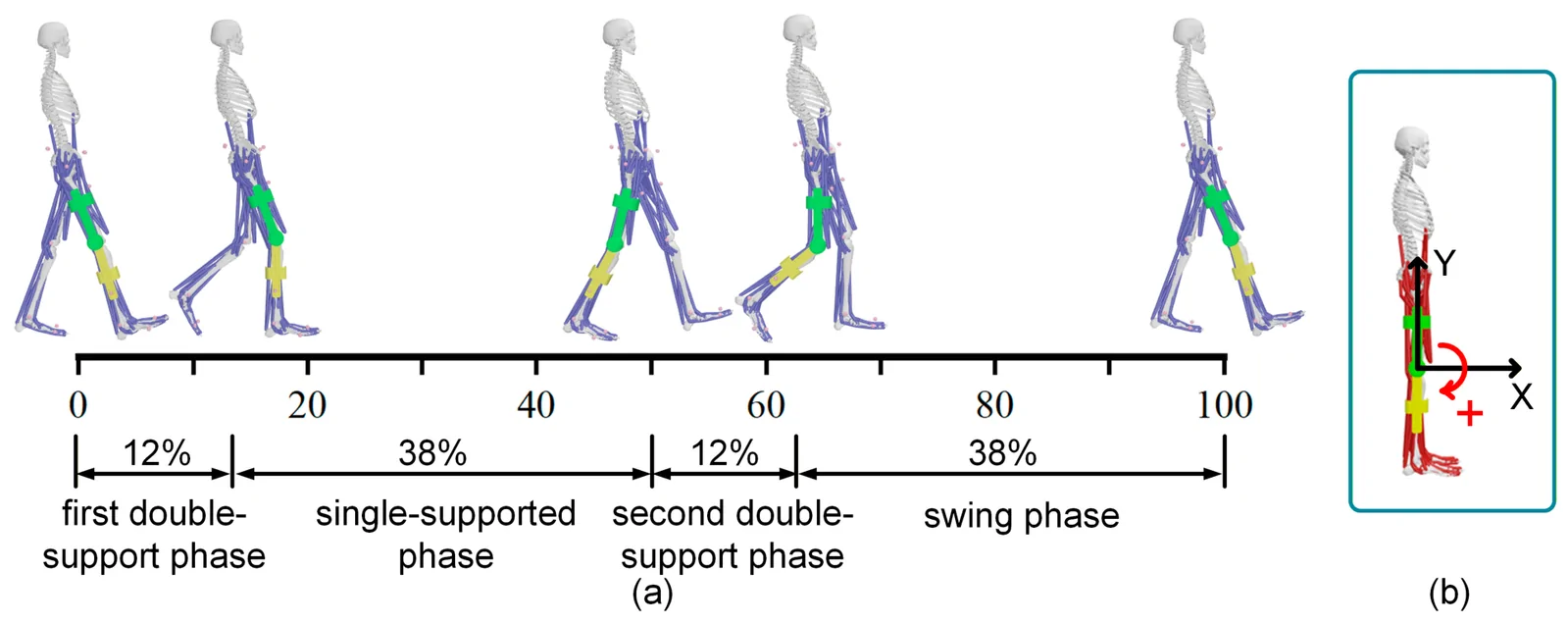
(**a**) Division of the human gait cycle; (**b**) sagittal plane knee joint coordinate axes.

In this study, using the human right leg as a reference, the gait cycle is divided proportionally into four phases, namely the first double-support phase, the single-support phase, the second double-support phase, and the swing phase [22], as shown in Figure 1a. The x-axis labels for the gait cycle phases in the figures of the results presented in Section 3 correspond to this division. Since the misalignment between the rotation axis of the exoskeleton’s knee joint and that of the human knee joint occurs primarily within the sagittal plane, this study considers only knee flexion and extension movements within the sagittal plane. As shown in Figure 1b, within the sagittal plane, the instantaneous center of rotation of the knee joint in the upright position is defined as the origin of the coordinate system, the horizontal forward direction is defined as the positive x-axis, the vertical upward direction is defined as the positive y-axis, and the clockwise direction is defined as the positive direction for knee joint angles and moments.

### 2.2. Human-Exoskeleton Coupling Modeling and Simulation Methods

Currently, OpenSim (Version 4.4. Stanford University) offers a variety of musculoskeletal models for studying human gait. Research by Roelker et al. [23] indicates that Gait2392 is the optimal model for studying the gait of healthy young adults. Therefore, this study selected the Gait2392 general model as a basis and added a knee exoskeleton model to construct a human-exoskeleton coupled model. Through dynamic simulation, we investigated the effect of misalignment of the exoskeleton’s knee rotation axis on the torque at the human knee joint. The OpenSim community and other database websites provide numerous publicly available experimental datasets, as well as related studies based on these datasets that examine different walking speeds and load conditions. This study utilized 3D motion capture data from 16 volunteers walking at a speed of 4 km/h, as reported in the study by Moreira et al. [25]. The anthropometric data for the 16 volunteers are shown in Table 1.

**Figure 2:**
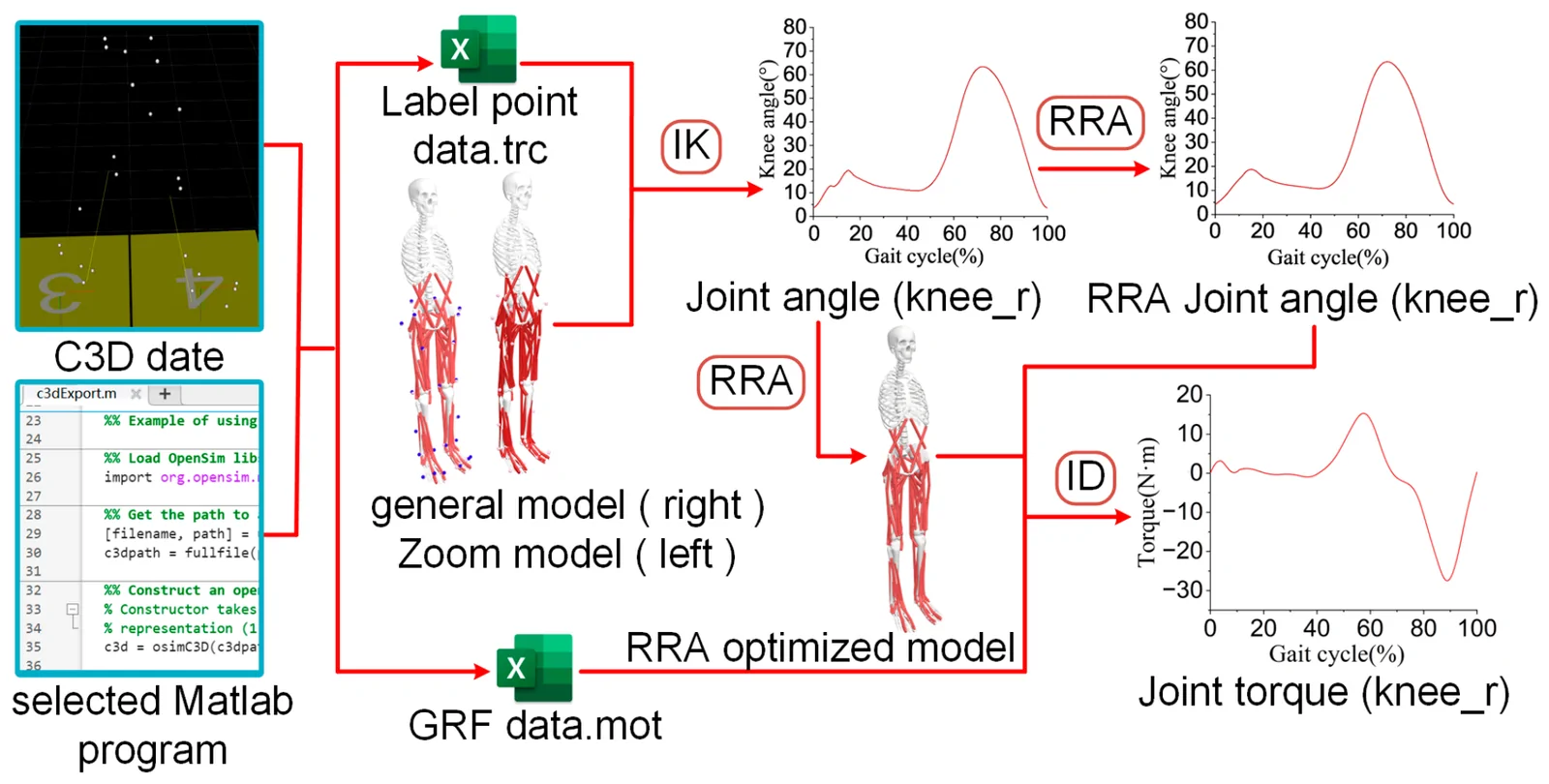
Process for constructing and simulating the musculoskeletal model.

The process for constructing and simulating the musculoskeletal model is shown in Figure 2. First, the Gait2392 general-purpose model was selected as the base template and linearly scaled based on the volunteers’ anthropometric data [26,27]. This was because the lower-limb degrees of freedom and muscle distribution of this model have been validated through extensive gait studies and can accurately reproduce the torque response of the knee joint in the sagittal plane while ensuring computational efficiency. Subsequently, a custom-developed MATLAB (Version 24.1.0.2537033 R2024a) program was used to convert the experimental C3D files into 3D marker point trajectories and ground reaction force (GRF) data, and joint angles were solved using inverse kinematics (IK) tools. It should be noted that no low-pass filtering was applied to the marker points during preprocessing, as the residual reduction process in subsequent steps would simultaneously optimize the kinematic data for noise, thereby avoiding signal peak attenuation caused by secondary filtering. Based on this, the Residual Reduction Algorithm (RRA) was applied to simultaneously optimize the human model’s mass, center of mass, and kinematic data [28,29] in order to eliminate residual external forces caused by marker point errors and modeling assumptions, thereby ensuring the physical consistency of the joint torques obtained from subsequent inverse dynamics (ID) calculations. Finally, the optimized kinematic data and the measured GRF data were input into the ID tool to obtain the human knee joint’s torques.

**Figure 3:**
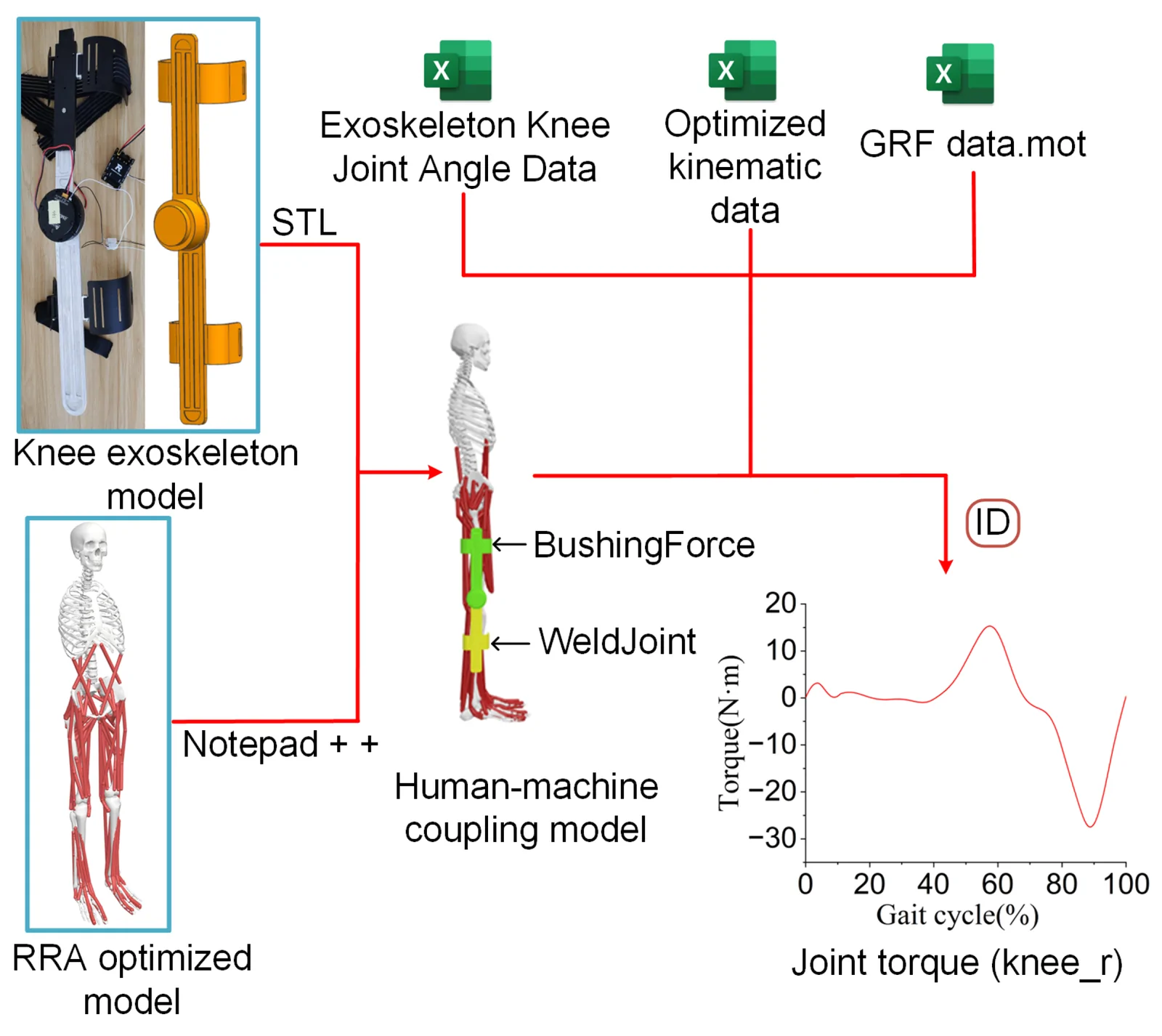
Human-exoskeleton coupling model construction process.

The process for constructing and simulating the human-exoskeleton coupling model is shown in Figure 3. To achieve precise parametric control of the misalignment degree, this study used a “Weld Joint” to rigidly connect the exoskeleton’s lower leg rod to the human lower leg bones, and quantitatively adjusted the misalignment by modifying the connection coordinates. This choice was made because the flexibility at the calf restraint site is significantly lower than that at the thigh, resulting in minimal relative displacement. Approximating this connection as rigid will neither introduce significant mechanical errors nor prevent the misalignment from being precisely locked at a preset value, thereby allowing for an independent assessment of the effect of misalignment on knee joint torque. Complementing this, spring stiffness and damping is applied at the thigh using the “Bushing Force” parameter to simulate the viscoelastic interaction forces of the restraint-soft tissue complex. The core of this approach lies in simultaneously accounting for the stiffness and damping of the restraint fabric and the stiffness and damping of the human lower limb’s muscles and soft tissues, which exhibit a series connection characteristic when loaded. To eliminate interference from the exoskeleton’s control strategy, this study directly uses the human knee joint motion angles obtained from the RRA as the exoskeleton’s drive signals, achieving the ideal state of “perfect tracking.” Although this assumption eliminates control errors, it allows the simulation results to be attributed solely to the single variable of mechanical misalignment; otherwise, any change in torque would be difficult to separate from the coupling between control and misalignment. The misalignment simulation was conducted along the positive and negative directions of the x and y axes in the human knee’s sagittal plane coordinate system, with an interval of 0.01 m and a total of 5 discrete points in each direction. This interval was selected on the basis of a preliminary sensitivity analysis: At smaller step sizes (e.g., 0.005 m), differences in the torque curves were no longer significant, whereas 0.01 m was sufficient to capture the gradual variation in torque from negligible to significant levels, while keeping the computational load manageable. Finally, the torque data from 16 volunteers under non-misalignment conditions and 20 misalignment conditions were averaged and normalized to obtain statistically significant representative curves.

Human-exoskeleton interaction forces and their configuration have always been a key focus of research on human-exoskeleton coupling simulations. Most current studies simulate human-exoskeleton interaction forces by applying spring stiffness and damping at the points where the exoskeleton is attached to the human body. The formula for calculating human-exoskeleton interaction forces is as follows:

(1) $$F=k\cdot \Delta x+C\cdot \dot{x}$$

Here, *k* represents the stiffness coefficient, *C* represents the damping coefficient, and *x* represents displacement. Stiffness and damping coefficients are typically set and adjusted based on empirical data and simulation results; there are no uniform regulations governing them. This study proposes an equivalent stiffness and damping configuration scheme that simultaneously accounts for both the exoskeleton’s attachment stiffness and damping and the human body’s stiffness and damping. Specifically, the exoskeleton’s attachment and the human body’s attachment points are treated as two springs connected in series. The formulas for calculating their equivalent stiffness *k_eq_* and equivalent damping *C_eq_* are as follows:

(2) $$\frac{1}{k_{eq}}=\frac{1}{k_{1}}+\frac{1}{k_{2}}$$

(3) $$\frac{1}{C_{eq}}=\frac{1}{C_{1}}+\frac{1}{C_{2}}$$

Here, *k*_1_ and *k*_2_ represent the stiffness of the bound body parts and the exoskeleton’s attachment points, respectively, while *C*_1_ and *C*_2_ represent the damping of the bound body parts and the exoskeleton’s attachment points, respectively.

The core assumptions of the abovementioned series equivalent model are as follows. (1) Both the soft tissues at the exoskeleton’s attachment points and the human body’s restrained regions are treated as massless, linear spring-damper elements, and the two are in a purely mechanical series relationship—that is, the normal force transmitted through the restraints acts entirely on the soft tissues, with no inertial effects from intermediate masses. (2) During loading, both the straps and the soft tissue satisfy small-deformation conditions, and their equivalent stiffness and damping are approximately constant within the load range of a single gait cycle. (3) Tangential slippage between the straps and the skin, as well as the resulting Coulomb friction, are neglected, and the normal interaction force is considered the primary load transfer path for human-exoskeleton coupling. These assumptions determine the model’s applicability limits: when the misalignment is small and the gait speed is moderate, soft tissue deformation remains in the approximately linear region, and the model’s predictions are relatively accurate. However, under conditions of large misalignment or high loads, the soft tissue may enter a nonlinear stiffening region, and the strap may exhibit gradually increasing stiffness due to fiber stretching; in such cases, the constant stiffness assumption will underestimate the peak interaction force; Furthermore, the model does not explicitly account for time-varying stiffness effects caused by active muscle contraction, nor does it reflect microslippage and shear forces at the bandage-skin interface. Therefore, the results of this study are most applicable to steady-state gait analysis under small to moderate misalignment conditions; conclusions should be extrapolated with caution for vigorous exercise or extreme misalignment conditions.

Since the physiological conditions vary among different subjects, factors such as muscle activation, joint angles, soft tissues’ properties, and the material, tightness, and orientation of the straps can all influence the calculation of equivalent stiffness and equivalent damping. Therefore, this study selected the mean values of stiffness and damping for the lower limb-exoskeleton attachment under normal conditions as a reference to calculate and set the equivalent stiffness and damping for human-exoskeleton coupling. Kang et al. estimated the in vivo viscoelastic properties of the human thigh under compression using a pendulum experiment [30], obtaining stiffness coefficients of 7500 N/m, 4500 N/m, 6000 N/m, and 4600 N/m, and damping coefficients of 150 N·s/m, 80 N·s/m, 130 N·s/m, and 80 N·s/m, respectively. Taking the average of these values yielded *k*_1_ = 5650 N/m and *C*_1_ = 110 N·s/m. The knee exoskeleton straps used in this study were made of nylon webbing 25 mm wide and approximately 1.5 mm thick. The tensile stiffness *k*_2_ was set to 300,000 N/m, and the damping coefficient *C*_2_ was set to 1000 N·s/m. Based on Equations (2) and (3), the equivalent stiffness *k_eq_* was calculated to be 5545.6 N/m, and the equivalent damping *C_eq_* was calculated to be 99.1 N·s/m.

### 2.3. Experiment on the Misalignment of the Rotation Axis of an Exoskeleton’s Knee Joint

**Figure 4:**
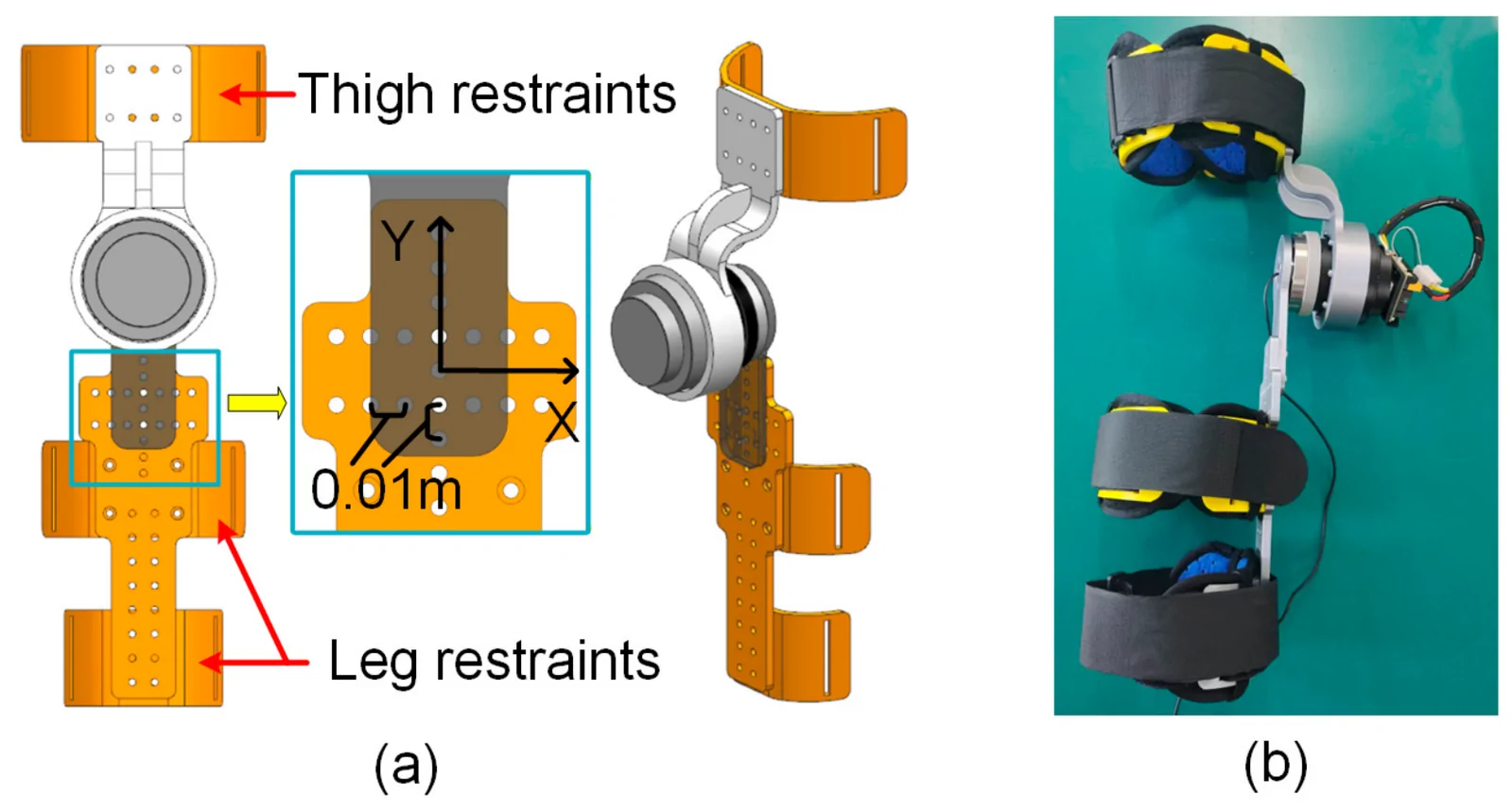
(**a**) Knee exoskeleton model; (**b**) knee exoskeleton prototype.

To facilitate experiments on the misalignment of the exoskeleton’s knee joint rotation axis, this study made several modifications to the knee exoskeleton. As shown in Figure 4, two attachment points were added to the exoskeleton’s lower leg rod to ensure a more secure fastening between the rod and the human lower leg. At the connection point between the exoskeleton’s lower leg rod and the knee joint’s drive rod, positioning holes were placed at 0.01 m intervals along the four directions of the coordinate axes. While ensuring a secure attachment between the exoskeleton’s lower leg rod and the human lower leg, connecting to different positioning holes allows for misalignment of the exoskeleton’s knee joint rotation axis by 0.01 m, 0.02 m, and 0.03 m along the four axes of the human knee joint’s sagittal plane coordinate system. The overall structure of the knee exoskeleton prototype was fabricated using 3D printing, which reduced the weight of the exoskeleton while ensuring structural strength.

The anthropometric data for the 8 volunteers are shown in Table 2. All volunteers were in good health and had no history of musculoskeletal or neurological disorders in the lower limbs. All volunteers provided written informed consent prior to the experiment. This study was reviewed and approved by the Ethics Committee of Qixia District Hospital, Nanjing, with approval number 2024-QX029.

The experimental procedure for assessing misalignment of the rotation axis of the exoskeleton’s knee joint is as follows.

**Figure 5:**
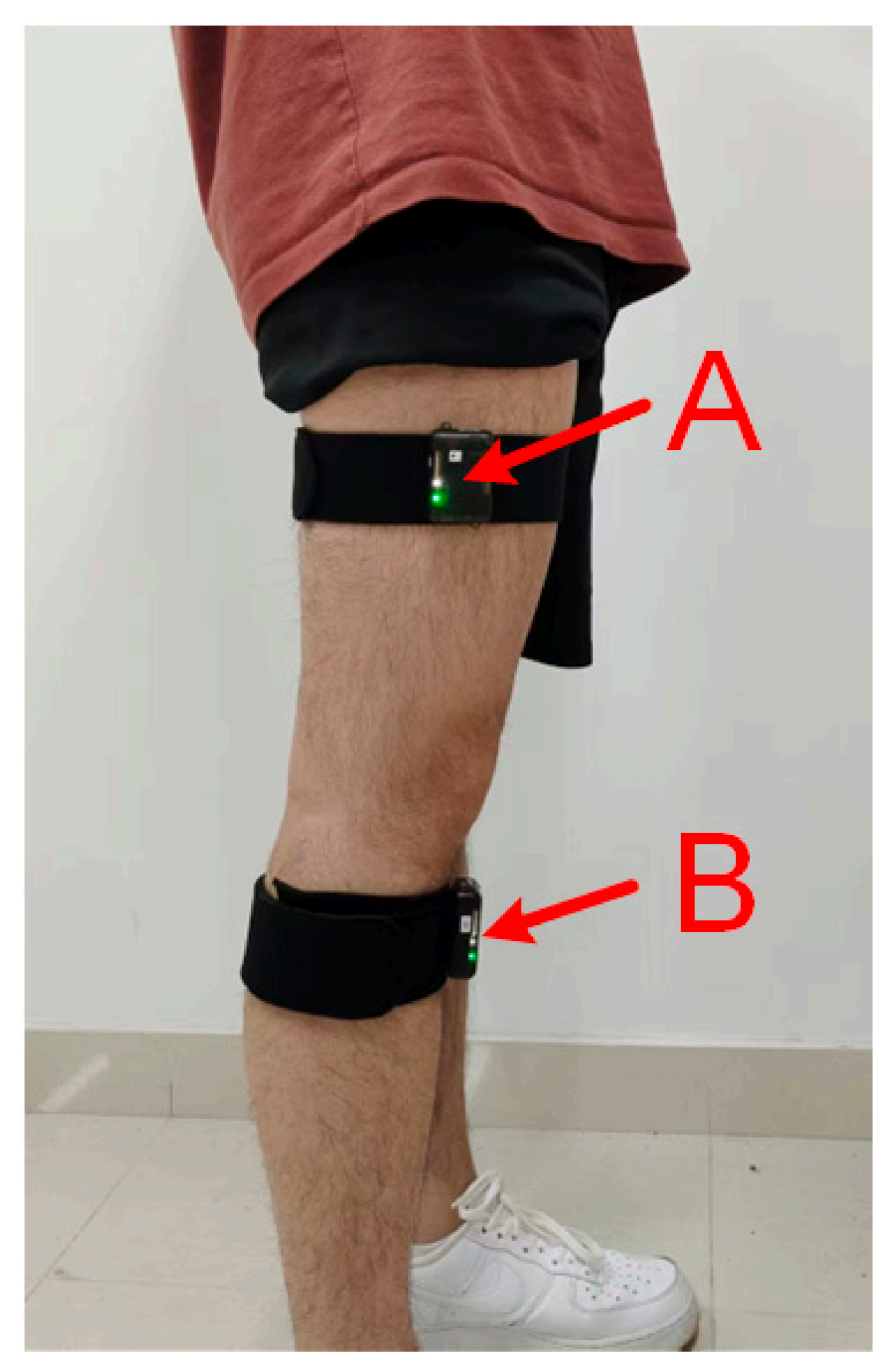
IMU location diagram. A: IMU on the thigh; B: IMU on the lower leg.

**Figure 6:**
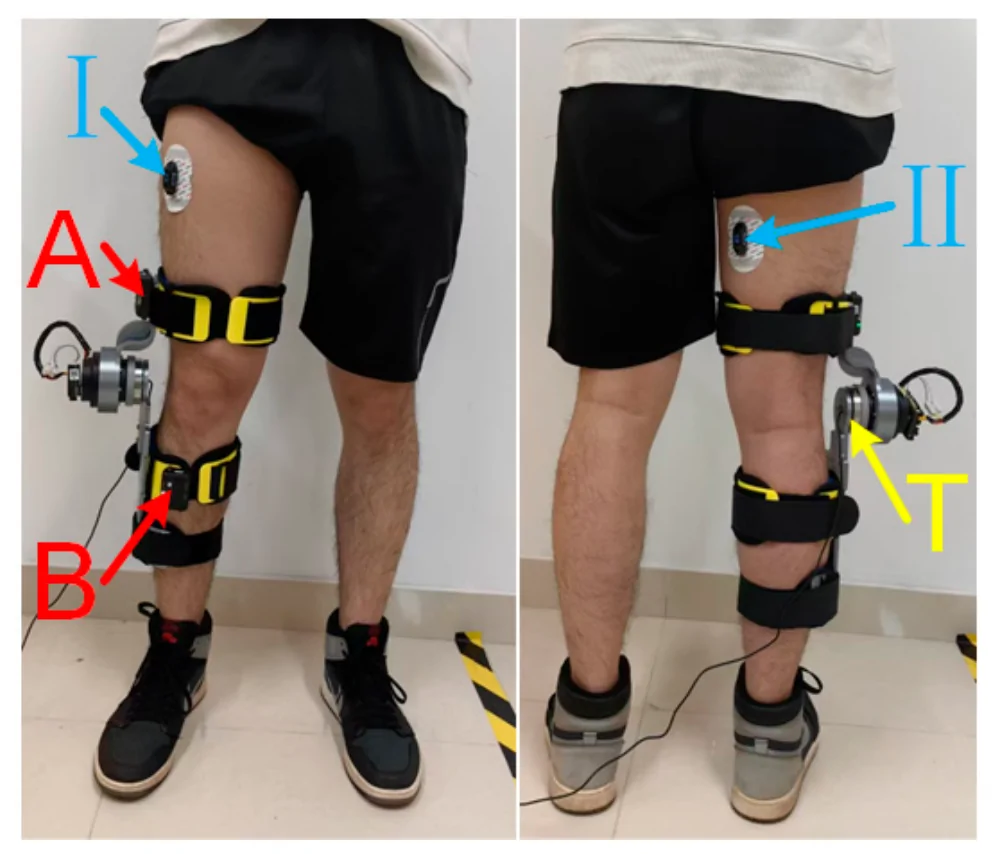
Sensors’ location. A: IMU on the thigh. B: IMU on the lower leg. I: Vastus lateralis. II: Biceps femoris. T: Exoskeleton’s knee torque sensor.

(1) An inertial measurement unit (IMU) (Track X inertial system. INFO. instruments Technology Co., Ltd. Shanghai, China) was attached to the corresponding locations on the volunteer’s right lower limb—the thigh and calf—as shown in Figure 5. Prior to each experiment, the IMU underwent static calibration: the sensor was placed on a level surface, and data were collected for 30 s to calculate the zero bias of the accelerometer and gyroscope. To ensure consistency in gait cycles, the volunteer walked at a normal gait in time with a metronome (0.75 s/beat). Each trial consisted of at least 3 complete gait cycles, with a total of 10 trials and a 5-min rest period between each trial. The data collected by the IMU were processed through the internal filters and solvers of the accompanying software to directly output knee joint angle data at a sampling frequency of 100 Hz.

(2) Volunteers wore the knee exoskeleton in a state of no misalignment, using the personalized knee angle data obtained for each volunteer in Step (1) as the target to drive the exoskeleton’s motors. Through manual alignment and adaptive walking while wearing the exoskeleton, the exoskeleton was brought into approximate alignment with the rotation axis of the human knee joint. IMUs were placed on the corresponding positions of the volunteers’ thighs and calves, and surface electromyography (EMG) (PICO. INFO. instruments Technology Co., Ltd. Shanghai, China) sensors were attached to the muscle bellies of the vastus lateralis and biceps femoris. Prior to attachment, hair was removed and the areas were cleaned with alcohol; the sensors’ locations are shown in Figure 6. During the human gait cycle, the primary muscles involved in knee flexion and extension include the vastus lateralis, vastus medialis, rectus femoris, and biceps femoris. Since the rectus femoris is a biarticular muscle, its activity is subject to interference from the hip joint’s movement during gait. Additionally, the EMG signals from the vastus medialis are less stable and weaker than those from the vastus lateralis. Therefore, this study selected the vastus lateralis and biceps femoris—which have higher and more stable EMG signal intensities—as the measurement targets. After electrode placement, maximum voluntary isometric contraction (MVC) calibration was performed. Volunteers performed 5-s maximum contractions of the vastus lateralis and biceps femoris, respectively, repeating this 10 times with a 2-min rest between sets; the mean of the maximum values from each trial was taken as the normalized EMG reference value for that muscle. The preprocessing steps for the raw EMG signals were as follows: bandpass filtering was used to remove motion artifacts and high-frequency noise; a notch filter was applied to eliminate power-line interference; after full-wave rectification, a low-pass filter was used to extract the linear envelope; finally, the result was divided by the corresponding muscle’s MVC reference value to obtain the muscle activation curve. The EMG sampling rate was 1000 Hz; during analysis, the EMG data were downsampled to 100 Hz to match the kinematic data.

(3) Volunteers walked at a normal gait for at least 10 complete gait cycles in time with the metronome (0.75 s/beat). A synchronizer was used to ensure that the IMU, EMG sensors, and torque sensors (LZ-NJY65. Hefei Lizhi Sensor Systems Co., Ltd. Hefei, China) recorded data simultaneously. Data from each sensor were recorded.
(4) We adjusted the misalignment of the exoskeleton’s knee joint rotation axis sequentially, repeated Step (3), and allowed a 5-min rest between groups.
(5) We compared the volunteer’s knee joint angle data with and without the knee exoskeleton. Experimental data showing significant discrepancies between the knee joint angle data with and without the exoskeleton were excluded, and the retained data were processed and analyzed.

## 3. Results

**Figure 7:**
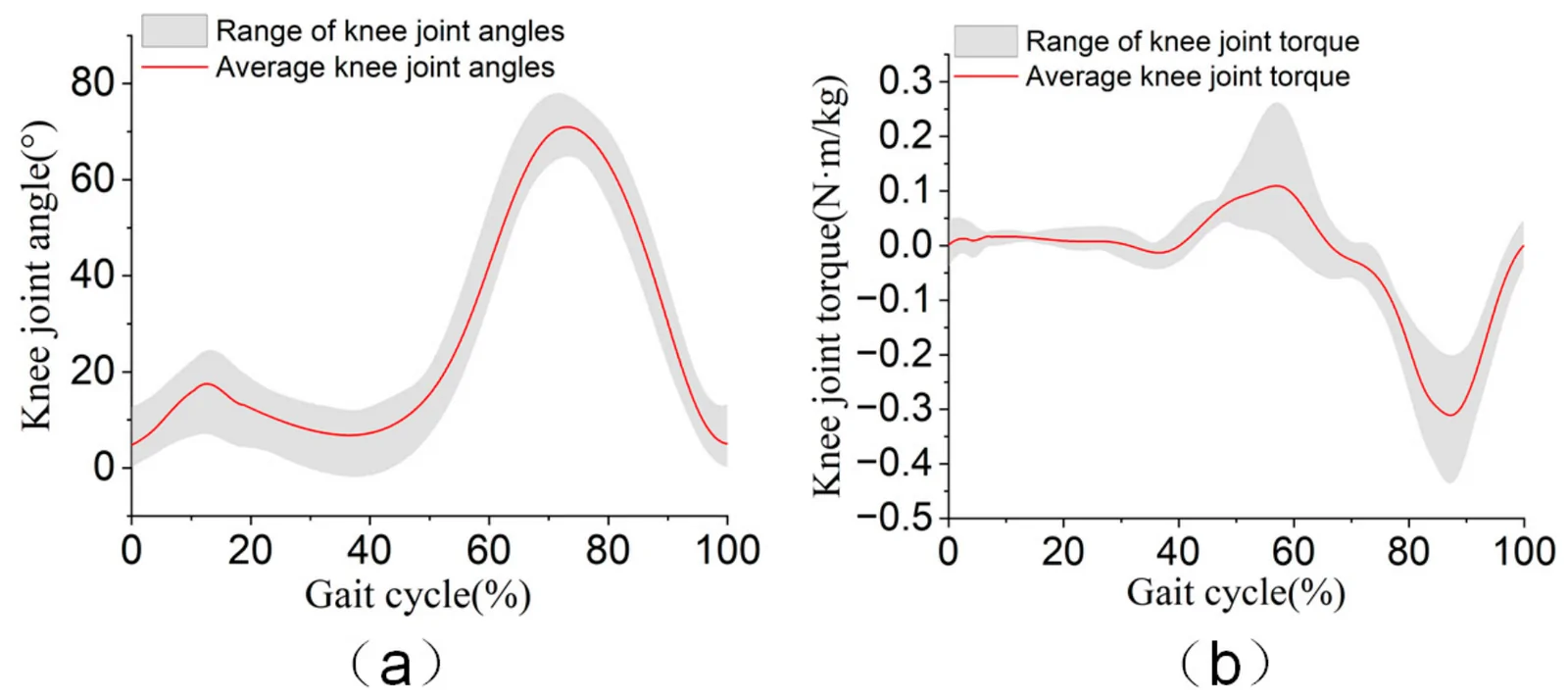
(**a**) Human knee joint angle; (**b**) human knee joint torque.

The knee angle and torque data obtained from the musculoskeletal model simulation were averaged and normalized to produce a curve representing the average values over one gait cycle, as shown in Figure 7. In Figure 7a, the knee angle reaches its first peak of 17.495° at 12.6% of the gait cycle, corresponding to the moment when the knee transitions from flexion to extension during the early single-support phase shown in Figure 1a; the second peak occurs at 73.1%, with an angle of 70.985°, corresponding to the transition from flexion to extension during the early swing phase. The knee joint torque curve in Figure 7b shows that between 0% and 40% of the gait cycle, the torque remains within a relatively low range of −0.013 N·m/kg to 0.017 N·m/kg, which is consistent with the gradual changes in knee joint angle during the first double-support phase and the single-support phase. The torque crosses zero at 66.5% of the gait cycle and changes direction, exhibiting a temporal difference relative to the second peak of the angle curve. This discrepancy stems from their differing mechanical meanings: the extrema of the angle curve correspond to instants when the joint’s angular velocity is zero, whereas the zero point of the torque curve corresponds to the instant when the net resultant torque is zero; the two need not be synchronized in non-quasi-static walking motion. During the swing phase, the inertial effect of the lower leg generates a passive extension torque as the knee extends; the extensor muscles must contract in advance to generate a flexion torque to counteract this, causing the net torque to cross zero and reverse direction before the angle reaches its peak. This temporal discrepancy between angle and torque is a mechanical manifestation of the combined effects of limb inertia and active muscular control; it also indirectly validates the physical consistency of inverse dynamics calculations in musculoskeletal models.

**Figure 8:**
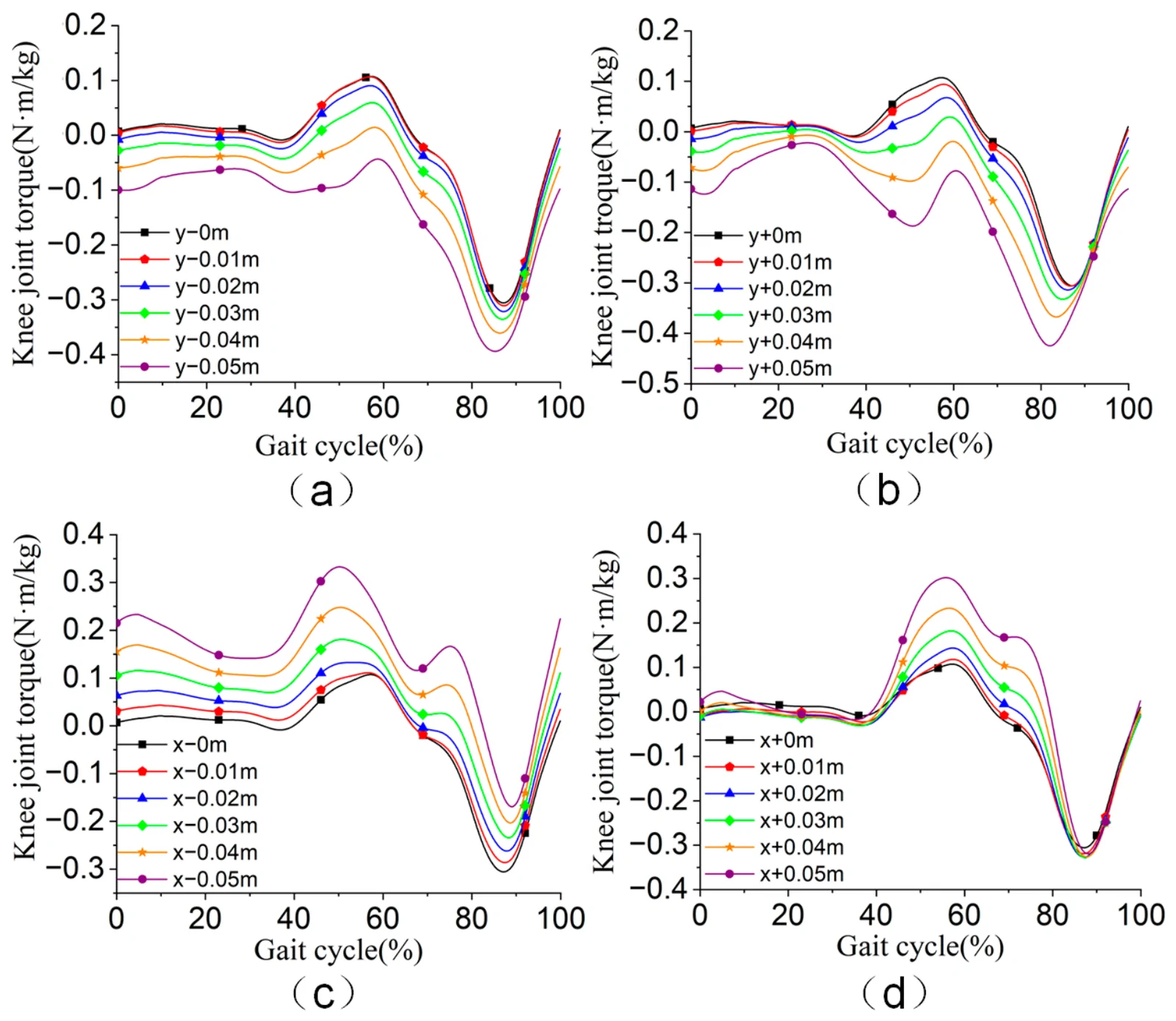
Curves showing changes in knee joint moments in the human knee under varying degrees of the exoskeleton’s knee joint rotation axis misalignment. (**a**) Misalignment in the negative direction of the Y-axis; (**b**) Misalignment in the positive direction of the Y-axis; (**c**) Misalignment in the negative direction of the X-axis; (**d**) Misalignment in the positive direction of the X-axis.

The knee joint torque data obtained from the human-exoskeleton coupling model simulation were averaged and normalized to produce a torque variation curve for different degrees of misalignment within a single gait cycle, as shown in Figure 8. The 0 m curve represents the reference condition with no misalignment of the rotation axis. Figure 8a shows misalignment in the negative y-axis direction: during the second double-support phase, the peak torque decreases from 0.107 N·m/kg (with no misalignment) to −0.043 N·m/kg (with 0.05 m misalignment), while the peak torque during the swing phase increases from −0.306 N·m/kg to −0.394 N·m/kg, representing a 28.8% increase in extension torque. The curves show an overall downward shift, and the underlying physical mechanism is as follows: when the exoskeleton’s knee joint experiences negative y-axis misalignment, its rotation axis generates a positive (flexion direction) offset lever arm relative to the human knee joint. The resulting human-exoskeleton interaction force drives lower leg flexion and impedes extension. The greater the misalignment, the more significant this additional torque becomes. Figure 8b shows misalignment in the positive y-axis direction: the torque curve also exhibits an overall downward shift, but the magnitude of the change is more pronounced. The peak value of the second double-support phase decreased from 0.107 N·m/kg to −0.078 N·m/kg; the peak value during the swing phase increased from −0.306 N·m/kg to −0.425 N·m/kg, with the extension torque increasing by 38.9%. The mechanism is similar to that of misalignment in the negative y-axis direction, also generating a positive human-exoskeleton interaction torque; however, the difference in the direction of the offset lever arm results in a more significant torque shift for the same degree of misalignment. Figure 8c shows misalignment in the negative x-axis direction: the torque curve exhibits an overall upward shift opposite to that in the y-axis direction. The peak value of the second double-support phase increased from 0.107 N·m/kg to 0.333 N·m/kg, representing a 211.2% increase in buckling torque, while the peak value in the swinging phase decreased from −0.306 N·m/kg to −0.169 N·m/kg. The underlying physical mechanism is as follows: when there is a misalignment of the exoskeleton’s axis of rotation in the negative x-axis direction, a counter-directional (extension direction) offset lever arm relative to the human knee joint is generated within the sagittal plane; the resulting interaction force drives lower leg extension and impedes flexion. Figure 8d shows misalignment in the positive direction of the x-axis: the effect of misalignment in this direction exhibits phase dependence. During the second double-support phase, the torque curve shifts upward, with the peak increasing from 0.107 N·m/kg to 0.302 N·m/kg, representing an 182.2% increase in flexion torque. The mechanism is similar to that of misalignment in the negative x-axis direction; however, during the swing phase, the change in peak torque across different misalignment levels is minimal (ranging from −0.306 N·m/kg to −0.328 N·m/kg), with no monotonic shift observed. This is because during the late swing phase of misalignment in the positive x-axis direction, the inertial forces of the exoskeleton’s lower leg rod and the sagittal component of the interaction forces partially offset each other, resulting in a negligible combined effect on the net torque at the knee joint. A comprehensive analysis of the misalignment results across the four directions reveals that misalignment along the y-axis primarily causes an overall downward shift in the torque curve, while misalignment along the x-axis is mainly manifested as a significant increase in torque during the stance phase; the swing phase is the least sensitive to misalignment in the positive x-axis direction. This difference stems from the varying coupling relationships between the misalignment directions and the relative degrees of freedom of motion between the exoskeleton and the human lower limb in the sagittal plane.

**Figure 9:**
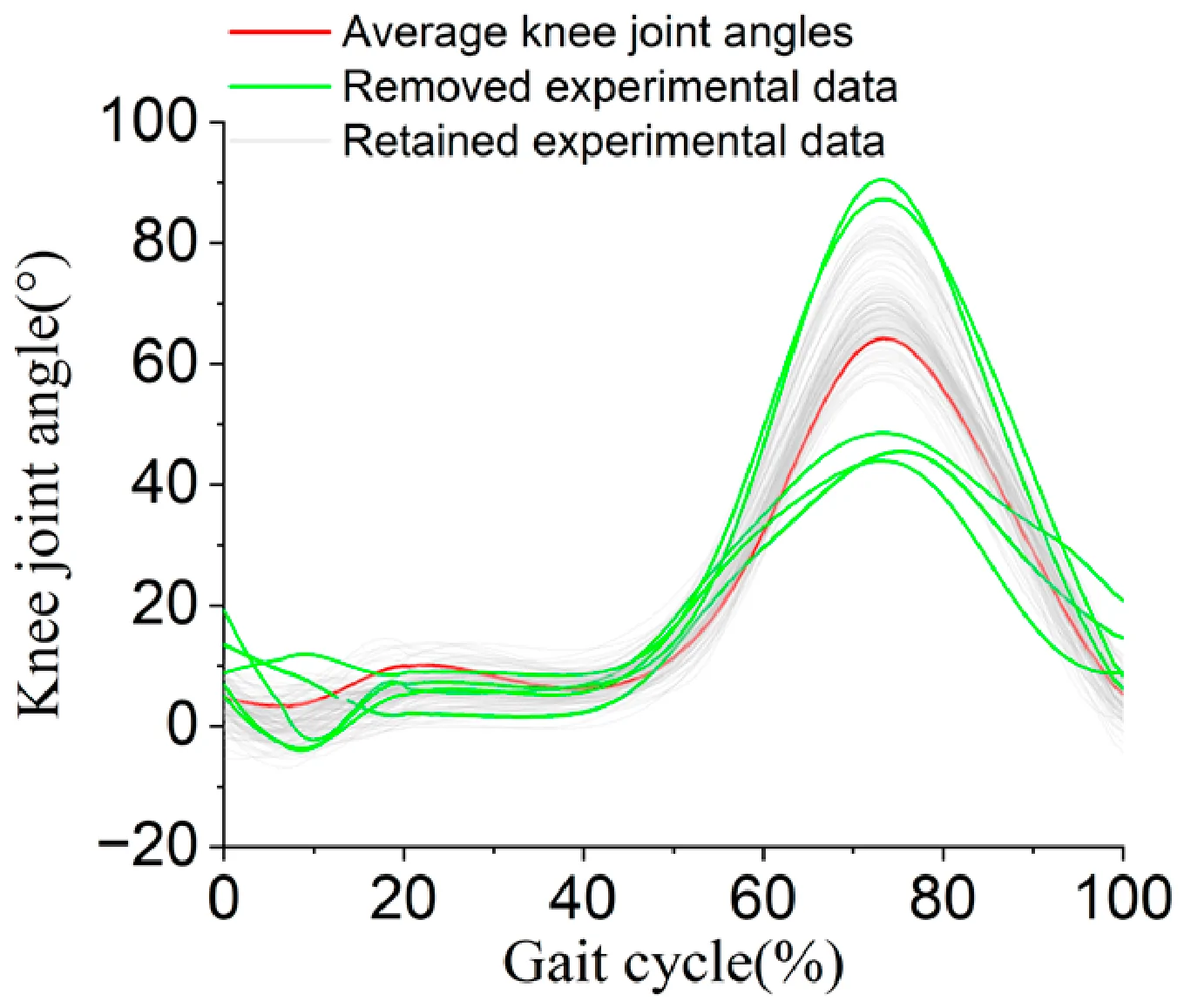
Comparison of knee angle curves for exoskeletons worn on and not worn on the human body.

Figure 9 compares the average knee angle curve (peak of 66.249°) for Volunteer P1 without the exoskeleton with the angle curves recorded while wearing the exoskeleton at different degrees of misalignment. Using ±30% of the average peak value when not wearing the exoskeleton as the tolerance range for normal gait (i.e., 46.374–86.124°), five abnormal curves that clearly exceeded this range were excluded; their peak values were 43.968°, 44.503°, 45.545°, 87.287°, and 90.529°. The corresponding torque sensor and EMG data were also removed simultaneously. Among these anomalous curves, excessively low peak values indicated that exoskeleton misalignment caused restricted knee flexion, resulting in a stiff gait pattern; conversely, excessively high peak values suggested that the exoskeleton was excessively pulling on the knee joint, which may have triggered a compensatory flexion strategy. The exclusion of the aforementioned data aims to eliminate the impact of severe misalignment interference and potential human-exoskeleton coupling instability on the reliability of the results, thereby retaining valid gait samples that truly reflect the effects of misalignment. Data from the remaining volunteers were processed according to the same criteria.

**Figure 10:**
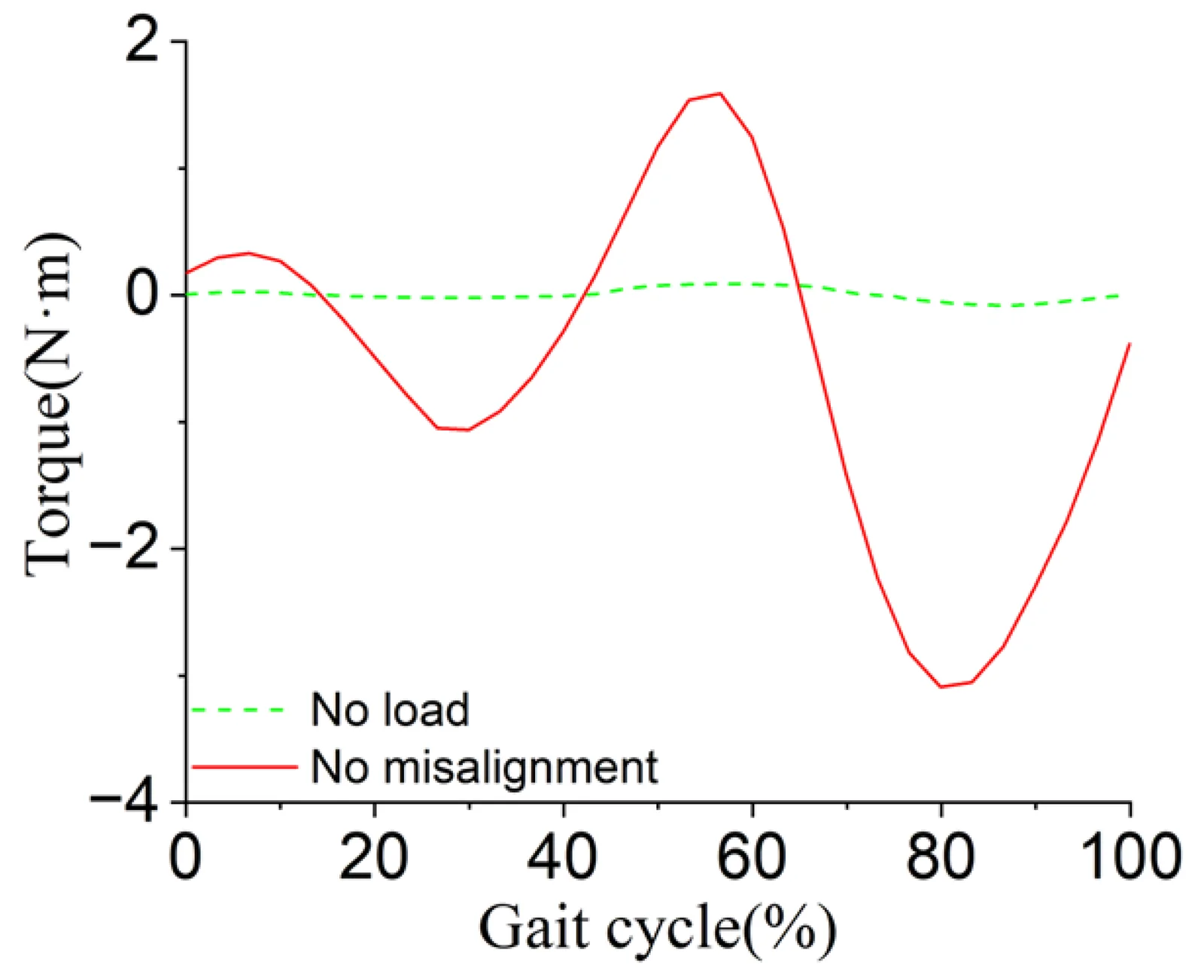
The torque curve measured by the torque sensor over one gait cycle.

Since the main body of the exoskeleton prototype was manufactured using 3D printing, it has a low self-weight, and the motor’s drive torque is very small during no-load operation. As shown in Figure 10, the maximum drive torque of the motor under no-load conditions is only 0.09 N·m, whereas the maximum torque measured by the sensor during walking with proper misalignment is 2.7 N·m; the former accounts for only 3% of the latter. Given that the motor’s drive torque is far smaller than the torque generated by human-exoskeleton interaction, in subsequent analyses, the measurements from the exoskeleton’s knee joint torque sensor will be approximated as the torque generated by human-exoskeleton interaction at the exoskeleton knee joint. Under steady-state walking conditions, the error introduced by this approximation does not exceed 3% and does not affect the assessment of the misalignment effect trend. Furthermore, under the assumption of an approximately rigid connection at the lower leg segment, this interactive torque can be regarded as equivalent to the external load torque applied by the exoskeleton to the human knee joint [31].

**Figure 11:**
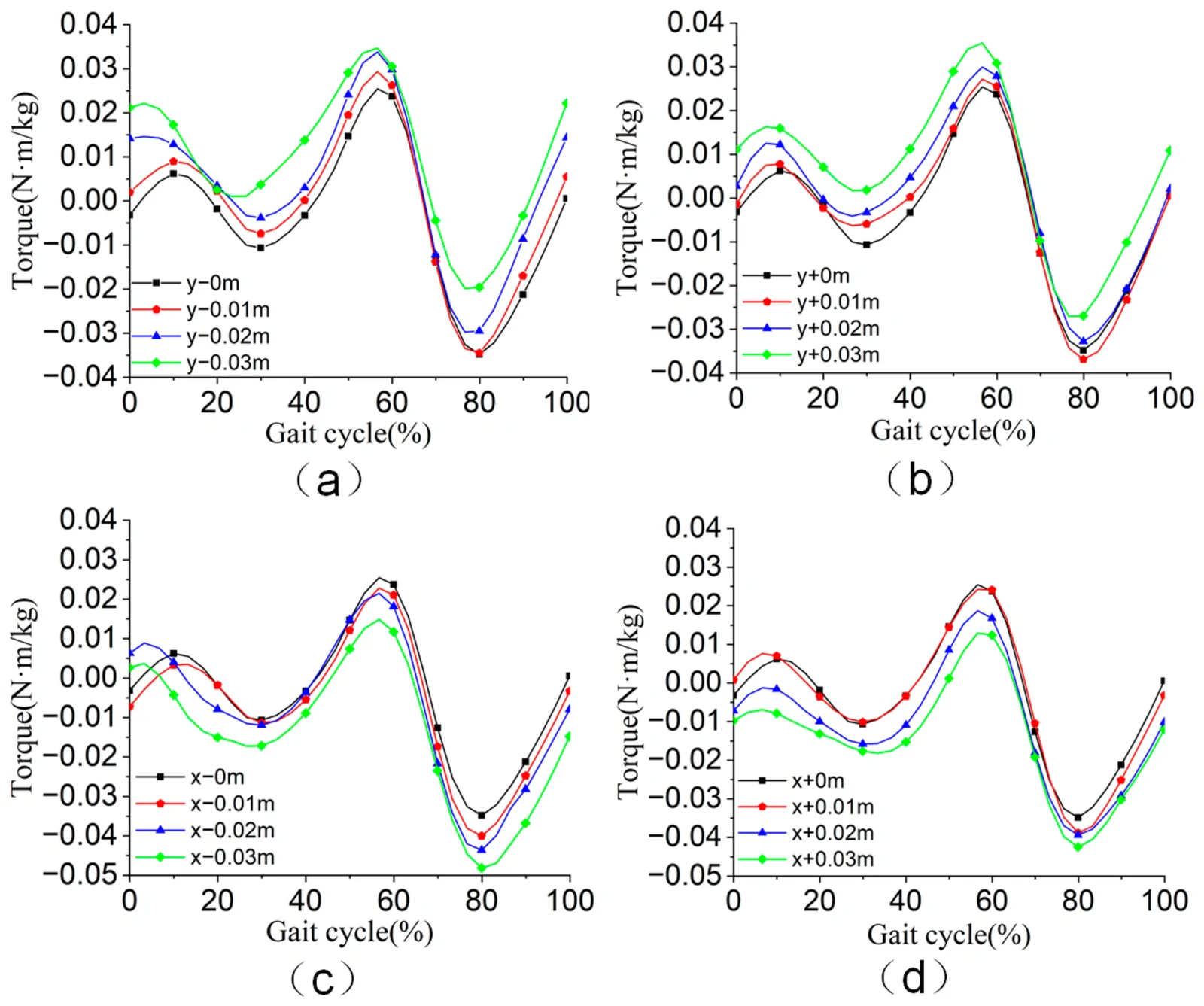
Curves showing changes in human-exoskeleton interaction forces at different degrees of misalignment during a single gait cycle. (**a**) Misalignment in the negative direction of the Y-axis; (**b**) Misalignment in the positive direction of the Y-axis; (**c**) Misalignment in the negative direction of the X-axis; (**d**) Misalignment in the positive direction of the X-axis.

After averaging and normalizing the torque data measured in the exoskeleton’s knee joint rotation axis misalignment experiment, we obtained human-exoskeleton interaction torque curves for a single gait cycle under different degrees of misalignment, as shown in Figure 11. Given the presence of uncontrollable interference factors in the experiment—such as the exoskeleton’s wearing adaptability and random gait fluctuations—the following analysis focuses on the overall trends in the torque curves rather than detailed comparisons of specific numerical values. Overall, the trends in the experimentally obtained human-exoskeleton interaction torque curves are consistent with those of the simulated knee joint torque curves (Figure 8), indirectly validating the accuracy of the human-exoskeleton coupling simulation results. Figure 11a shows misalignment in the negative y-axis direction: the peak torque during the second double-support phase increases from 0.025 N·m/kg to 0.035 N·m/kg, indicating that as misalignment increases, the positive interactive torque during this phase becomes stronger. This precisely compensates for the positive torque demand of the human knee joint, causing it to decrease, as shown in Figure 8a. The peak value in the swing phase decreases from −0.035 N·m/kg to −0.020 N·m/kg, implying that the forward interactive torque during the extension phase weakens as misalignment increases, forcing the human body to generate a larger forward torque to maintain gait-corresponding to the increase in forward torque with misalignment shown in Figure 8a. Figure 11b shows misalignment in the positive y-axis direction: the peak torque during the second double-support phase increases from 0.025 N·m/kg to 0.035 N·m/kg, indicating that misalignment in this direction also provides a gradually increasing positive interactive torque during the flexion phase, causing the body’s positive torque to decrease, as shown in Figure 8b. The peak value in the swing phase decreases from −0.035 N·m/kg to −0.027 N·m/kg; the counteracting torque weakens, requiring the human body to increase its counteracting torque output, consistent with the pattern shown in Figure 8b. Figure 11c shows misalignment in the negative x-axis direction: contrary to the y-axis direction, the peak torque in the second double-support phase increases from 0.025 N·m/kg to 0.015 N·m/kg; that is, the forward interaction torque weakens with misalignment, leading to a corresponding increase in the body’s demand for forward torque, as shown in Figure 8c. The peak value of the swing phase increases from −0.035 N·m/kg to −0.048 N·m/kg, indicating an increase in the reverse interaction torque. At this point, the body only needs to generate a small reverse torque to complete the extension, which is consistent with the trend of decreasing reverse torque shown in Figure 8c. Figure 11d shows misalignment in the positive x-axis direction: the pattern is consistent with that in the negative x-axis direction but with a slightly different amplitude. The peak value of the second double-support phase decreases from 0.025 N·m/kg to 0.013 N·m/kg; the forward interaction torque weakens, and the forward torque required by the body increases, corresponding to Figure 8d. The peak value in the swing phase increases from −0.035 N·m/kg to −0.042 N·m/kg; the reverse interaction torque increases, while the human body’s demand for reverse torque decreases, consistent with the trend shown in Figure 8d. A comparison of these trends indicates that the variation patterns of the human-exoskeleton interaction torque measured in the experiment, as a function of the direction and degree of misalignment, are complementary to the changes in human knee joint torque calculated by the simulation. This systematic correspondence in both timing and amplitude effectively validates, from a secondary perspective, the physical plausibility of the established human-exoskeleton coupling model and its simulation results.

**Figure 12:**
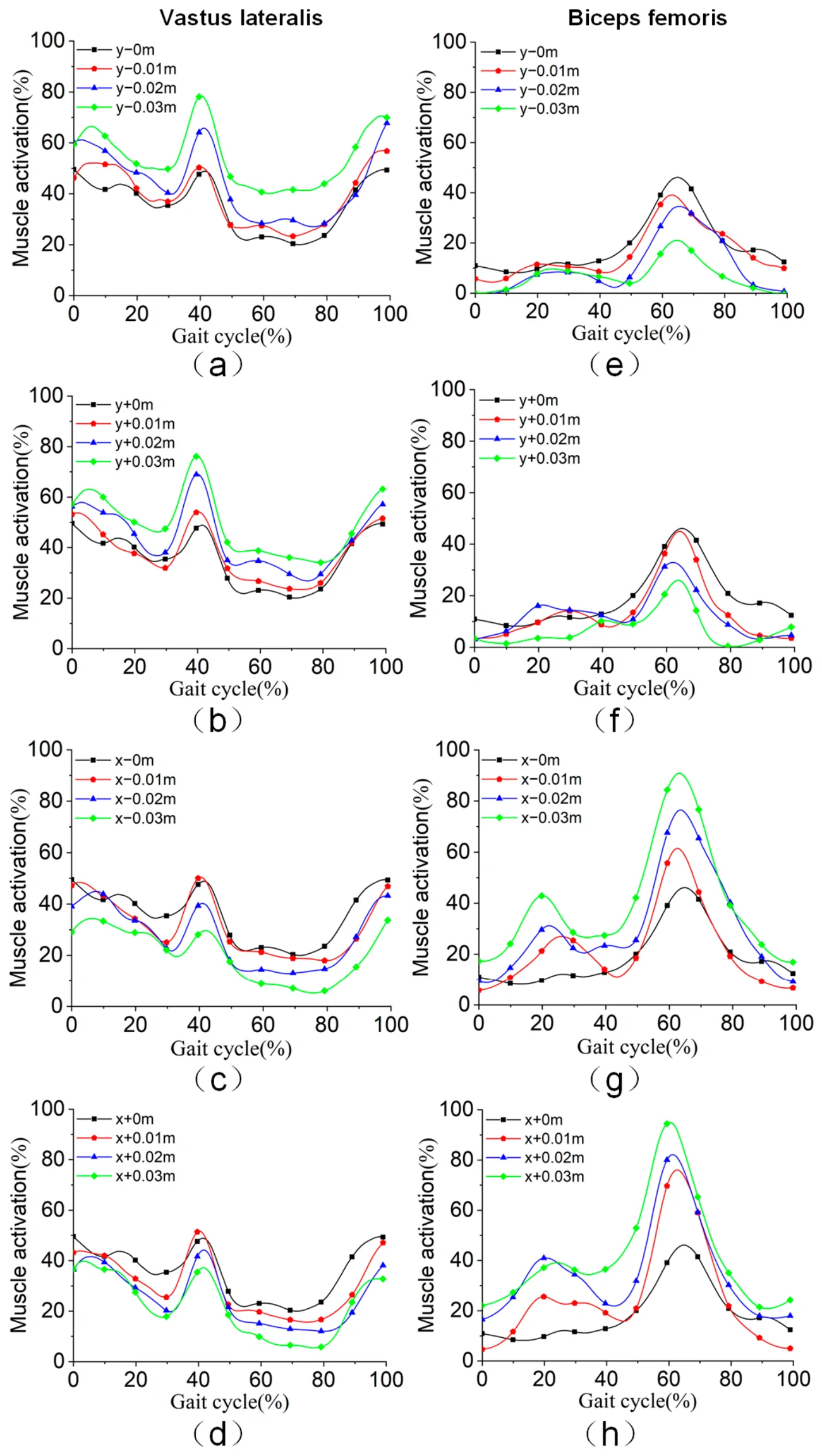
(**a**) Vastus lateralis muscle activation at different degrees of misalignment in the negative direction of the Y-axis; (**b**) Vastus lateralis muscle activation at different degrees of misalignment in the positive direction of the Y-axis; (**c**) Vastus lateralis muscle activation at different degrees of misalignment in the negative direction of the X-axis; (**d**) Vastus lateralis muscle activation at different degrees of misalignment in the positive direction of the X-axis; (**e**) Biceps femoris muscle activation at different degrees of misalignment in the negative direction of the Y-axis; (**f**) Biceps femoris muscle activation at different degrees of misalignment in the positive direction of the Y-axis; (**g**) Biceps femoris muscle activation at different degrees of misalignment in the negative direction of the X-axis; (**h**) Biceps femoris muscle activation at different degrees of misalignment in the positive direction of the X-axis.

After preprocessing the raw EMG signals collected during the misalignment experiments—including filtering, denoising, and full-wave rectification—the integrated EMG values were calculated and normalized to obtain the average activation curves of the vastus lateralis and biceps femoris muscles over one gait cycle at different degrees of misalignment, as shown in Figure 12. The direction of change in muscle activation is determined by the polarity of the human-exoskeleton interaction torque and forms a functionally complementary correspondence with the simulated knee joint torque (Figure 8). Figure 12a,b,e,f show misalignment along the y-axis: in this case, the interaction torque is positive (assisting flexion and resisting extension). As the misalignment increases, the vastus lateralis, which is responsible for extension, must overcome progressively greater interactive resistance; its peak activation level rises from 48.9% (no misalignment) to 78.5% (Y−) and 76.1% (Y+); conversely, the biceps femoris, responsible for flexion, benefits from the assisting interactive torque, causing its peak activation to decrease from 46.1% to 21.0% (Y−) and 26.0% (Y+). This pattern of changes aligns with the trend observed in Figure 8a,b, where the extension torque of the human knee joint increases while the flexion torque decreases. Figure 12c,d,g,h show misalignment along the x-axis: in this case, the interaction torque is in the opposite direction (assisting extension, hindering flexion). To perform flexion against the interactive resistance, the biceps femoris saw its peak activation rise from 46.1% to 90.9% (X−) and 95.0% (X+); conversely, the vastus lateralis, aided by the interactive torque, saw its peak activation decrease from 48.9% to 29.6% (X−) and 37.2% (X+). This is consistent with the pattern shown in Figure 8c,d, where flexion torque increases and extension torque decreases. The muscle activation patterns obtained from the experiment corroborate the human-exoskeleton coupled simulation torques in terms of both magnitude and timing, further validating the accuracy of the simulation results from the perspective of neuromuscular control.

Based on the verified accuracy of the human-exoskeleton coupling simulation results, further processing and analysis of these results yield the data listed in Table 3. The Maximum Difference Ratio (MDR) is the ratio of the maximum difference in torque at the human knee joint between conditions of misalignment and non-misalignment to the peak torque under non-misalignment conditions. The Assist Efficiency (AE) is the ratio of the difference in work performed by the human knee joint torque during one gait cycle under conditions of misalignment and non-misalignment to the work performed under non-misalignment conditions, that is

(4) $$AE=\frac{W_{1}-W_{2}}{W_{1}}$$

Here, *W*_1_ represents the work performed by the torque at the human knee joint during one gait cycle under non-misalignment conditions, and *W*_2_ represents the work performed by the torque at the human knee joint during one gait cycle under misalignment conditions. The formula for work performed by torque is

(5) W=∫|τ|dθ=∫|τ(t)|⋅ω(t)dt

Here, *τ* represents torque, *θ* represents the knee joint angle, *ω* represents the angular velocity of the knee joint, and *t* represents time. Based on the knee joint angle and torque curve data from the human-exoskeleton coupling simulation described above, the angular velocity is calculated using numerical differentiation:

(6) $$\omega (t_{i})=\frac{\left|\theta (t_{i+1})-\theta (t_{i-1})\right|}{t_{i+1}-t_{i-1}}$$

(7) $$\omega (t_{0})=\frac{\left|\theta (t_{1})-\theta (t_{0})\right|}{t_{1}-t_{0}}$$

(8) $$\omega (t_{n})=\frac{\left|\theta (t_{n})-\theta (t_{n-1})\right|}{t_{n}-t_{n-1}}$$

Here, *ω*(*t*_0_) is the angular velocity at the starting point, and *ω*(*t_n_*) is the angular velocity at the ending point. Assuming a constant time interval Δ*t* = *t*_*i*+__1_ − *t_i_*, the work performed by the torque on the human knee joint during one gait cycle is

(9) $$W=\Delta t\left[\frac{\left|\tau (t_{0})|\cdot \omega (t_{0})+|\tau (t_{n})|\cdot \omega (t_{n}\right)}{2}+\sum_{i=1}^{n-1}\left|\tau (t_{i})|\cdot \omega (t_{i}\right)\right]$$

We can calculate *W*_1_ and *W*_2_ using Equation (9), then substitute these values into Equation (4) to calculate *AE*.

As shown in Table 3, when the exoskeleton’s knee joint rotation axis experiences a misalignment of 0.01 m along each of the four axes of the knee joint coordinate system, the maximum relative differences are 5.69%, 17.82%, 22.16%, and 13.4%, respectively, while the exoskeleton’s assistive efficiency is −0.55%, 0.49%, 0.25%, and −1.89%, respectively. This indicates that when the misalignment between the exoskeleton’s and the human knee joints’ rotational axes is less than 0.01 m, the human-exoskeleton interaction forces exerted by the exoskeleton have a minimal impact on the human knee joint torque, do not interfere with the wearer’s normal walking gait, and have little effect on the exoskeleton’s assistive efficiency; When the misalignment of the exoskeleton’s knee joint rotation axis is 0.02 m, there is a relatively significant difference between the human knee joint torque curve and the knee joint torque curve when the exoskeleton’s rotation axis is not misaligned, with maximum difference ratios of 17.62%, 52.18%, 54.65%, and 34.88%. The exoskeleton’s assistive efficiency is −1.69%, −0.41%, −0.93%, and −5.13%. This indicates that when the misalignment between the exoskeleton’s and the human knee joint’s rotational axes reaches 0.02 m, the human-exoskeleton interaction forces have a significant impact on the human knee joint torque, and misalignment in the positive x-axis direction leads to a marked decrease in the exoskeleton’s assist efficiency; When the misalignment of the exoskeleton’s knee joint rotation axis reaches 0.03 m, the maximum percentage differences between the human knee joint torque curves and those when the exoskeleton’s rotation axis is not misaligned are 48.09%, 103.59%, 99.88%, and 71.98%. The exoskeleton’s assistive efficiency is −6.07%, −15.9%, −9.64%, and −13.54%. The maximum deviations in the positive y-axis and negative x-axis directions are nearly double the peak values of the human knee joint torque curve when the exoskeleton’s rotation axis is properly aligned, which would have a severe impact on the wearer’s knee joint torque. Furthermore, misalignment in any direction leads to a significant decrease in the exoskeleton’s assistance efficiency. The impact on the human knee torque curve and the exoskeleton’s assistance efficiency becomes even more severe when the misalignment reaches 0.04 m and 0.05 m. If the effects of the exoskeleton’s own weight and control program errors are also considered, the maximum difference between the human knee joint torque curve and the torque curve when the exoskeleton’s rotation axis is not misaligned will be even greater under the same degree of misalignment, and the impact on the exoskeleton’s assistance efficiency will also be greater. Therefore, during the exoskeleton’s structural design and human-exoskeleton coupling simulation, it is essential to ensure that the misalignment between the exoskeleton and the human knee joint’s rotation axis along the four axes of the knee joint coordinate system is less than 0.02 m.

## 4. Discussion

Under actual wearing conditions, due to the exoskeleton’s own weight and issues with its fastening structure, the rotation axis of the exoskeleton knee joint is more prone to misalignment in the negative direction of the y-axis in the knee joint coordinate system. Therefore, when designing the exoskeleton structure, we also focus on preventing the rotation axis of the exoskeleton’s knee joint from experiencing misalignment in the negative direction of the y-axis in the knee joint coordinate system. As shown in Figure 8 and the data above, for the same degree of misalignment, misalignment in the negative y-axis direction results in the smallest proportion of the difference in human knee joint torque curves. For example, at a misalignment of 0.03 m, the maximum difference in the negative y-axis direction accounts for 48.09%, while the difference in the positive y-axis direction accounts for 103.59%. This indicates that, compared with other directions, a misalignment of the exoskeleton’s knee joint rotation axis in the negative y-axis direction has a smaller impact on the human knee joint torque.

As shown in Figure 8a, the human-exoskeleton interaction force resulting from the misalignment of the exoskeleton’s knee joint rotation axis in the negative y-direction generates a positive torque at the human knee joint. This causes the human knee joint torque curve to shift downward overall, increasing the reverse torque at the knee joint during the knee extension phase in the late swing phase. Whereas, during the knee flexion phases of the second double-support phase and the early swing phase, it actually provides an assistive effect on the human knee joint, reducing the positive torque at the human knee joint. As shown in Figure 8b and the data above, the misalignment of the exoskeleton’s knee joint rotation axis in the positive direction of the y-axis has a significant impact on the human knee joint torque. The resulting human-exoskeleton interaction force also generates a positive torque at the human knee joint; however, compared with misalignment in the negative direction of the y-axis, the exoskeleton’s assistance efficiency is 0.49% when the misalignment is 0.01 m. A slight misalignment of the exoskeleton’s knee joint rotation axis in the negative direction of the y-axis actually leads to an increase in the exoskeleton’s assistance efficiency throughout the entire gait cycle. This is because the assistive effect of the exoskeleton’s knee joint rotation axis misalignment during the knee flexion phase outweighs the resistive effect it exerts on the human knee joint during the knee extension phase. As shown in Figure 8c and the data above, the human-exoskeleton interaction force caused by the misalignment of the exoskeleton’s knee rotation axis in the negative x-axis direction generates a counteracting torque at the human knee joint. This causes the human knee torque curve to shift upward overall. During the knee extension phase in the late swing phase, this reduces the torque on the human knee joint, providing a certain degree of assistance, whereas in other phases, it increases the human knee joint torque. At a misalignment of 0.01 m, the change in the exoskeleton’s assistance efficiency is 0.25%, which is also because the assistive effect of the exoskeleton’s knee joint rotation axis misalignment on knee extension outweighs the hindering effect on other phases. As shown in Figure 8d, the human-exoskeleton interaction force caused by the misalignment of the exoskeleton’s knee joint rotation axis in the positive direction of the x-axis also generates a counteracting torque at the human knee joint, resulting in an overall upward shift in the human knee joint torque curve. However, unlike the case in the negative direction of the x-axis, its effect on human knee joint torque during the knee extension phase is not significant, whereas during the knee flexion phase, it significantly increases the positive torque at the human knee joint. Therefore, the misalignment of the exoskeleton’s knee rotation axis in the positive direction of the x-axis only increases the torque at the human knee joint and should be strictly avoided in exoskeleton design.

This study currently only considers the effects of exoskeletal knee rotation axis misalignment along the four axes of the human knee coordinate system in the sagittal plane on human knee moments and exoskeletal assistance efficiency. Future research should expand the scope to investigate the effects of exoskeletal knee rotation axis misalignment within the four quadrants of the human knee coordinate system in the sagittal plane on human knee moments, thereby refining the data. To quantitatively analyze the effects of misalignment of the exoskeleton’s rotation axis on human knee joint moments, this study employed human-exoskeleton coupling simulations using multiple fixed misalignment points in different directions. However, in actual use, the direction and magnitude of the exoskeleton’s rotation axis misalignment continuously vary throughout the entire gait cycle. Therefore, the results of this study are applicable to the quantitative analysis of the effects of exoskeletal knee rotation axis misalignment on human knee joint moments and exoskeletal assistive efficiency, and can be used to guide and improve the structural design of knee exoskeletons.

## 5. Conclusions

This study established a human-exoskeleton coupling model capable of precisely adjusting the position of the exoskeleton’s knee joint rotation axis. The proposed equivalent series spring stiffness-damping scheme upgrades the simulation of human-exoskeleton interaction forces from an empirical approach to a physical model, providing a methodological foundation for the quantitative assessment of misalignment effects. The effect of misalignment of the exoskeleton’s knee joint rotation axis on human knee joint torque exhibits significant directional and phase dependencies. Misalignment in the y-axis direction primarily causes an increase in extension torque during the swing phase; when misaligned by 0.05 m in the positive y-axis direction, the peak value increases from −0.306 N·m/kg to −0.425 N·m/kg. Misalignment along the x-axis causes a sharp increase in the flexion torque during the stance phase; when there was a misalignment of 0.05 m in the negative x-axis direction, the peak value increased from 0.107 N·m/kg to 0.333 N·m/kg. The human-exoskeleton interaction torques measured experimentally were complementary to the simulated torques, indirectly validating the physical plausibility of the simulation model. During y-axis misalignment, the activation level of the vastus lateralis increased from 48.9% to 78.5% (Y−) and 76.1% (Y+), while that of the biceps femoris decreased from 46.1% to 21.0% (Y−) and 26.0% (Y+). When there was misalignment of the x-axis, the activation of the biceps femoris increased from 46.1% to 90.9% (X−) and 95.0% (X+), while that of the vastus lateralis decreased from 48.9% to 29.6% (X−) and 37.2% (X+). These changes were consistent with the direction of the interaction torque, further corroborating the simulation results from a physiological perspective. The maximum difference ratios in torque at a combined misalignment of 0.02 m were 17.62% in the negative y-axis direction, 52.18% in the positive y-axis direction, 54.65% in the negative x-axis direction, and 34.88% in the positive x-axis direction. Changes in auxiliary efficiency were −1.69% in the negative y-axis direction, −0.41% in the positive y-axis direction, −0.93% in the negative x-axis direction, and −5.13% in the positive x-axis direction; 0.02 m was determined to be the acceptable upper limit for misalignment. This quantitative criterion provides a direct basis for the design of knee exoskeleton joint structures and wearer calibration.

## Figures and Tables

**Table 1:** Physical measurements of 16 volunteers.

ID	Gender	Age (Years)	Body Height (m)	Body Mass (kg)	Leg Length (m)
1	Male	25	1.81	66.2	0.84
2	Female	23	1.56	54.4	0.69
3	Female	26	1.57	53.5	0.70
4	Male	23	1.71	81.7	0.79
5	Male	24	1.79	74.4	0.82
6	Female	21	1.65	60.0	0.74
7	Female	21	1.54	58.4	0.66
8	Male	23	1.72	75.8	0.77
9	Male	26	1.80	83.7	0.81
10	Male	25	1.82	76.8	0.81
11	Female	23	1.51	52.0	0.67
12	Male	25	1.83	73.4	0.82
13	Female	27	1.61	53.5	0.70
14	Female	26	1.60	65.3	0.71
15	Female	20	1.70	69.3	0.75
16	Male	22	1.79	81.9	0.79

**Table 2:** Physical measurements of eight volunteers.

ID	Gender	Age (Years)	Body Height (m)	Body Mass (kg)	Leg Length (m)
P1	Male	26	1.80	75.2	0.81
P2	Female	26	1.73	64.4	0.76
P3	Male	23	1.90	90.7	0.91
P4	Male	25	1.71	74.3	0.77
P5	Male	26	1.78	81.5	0.80
P6	Female	24	1.68	58.6	0.73
P7	Male	24	1.84	76.4	0.83
P8	Female	23	1.64	55.1	0.70

**Table 3:** MDR and AE at different degrees of misalignment.

Misalignment	Y−		Y+		X−		X+	
	MDR	AE	MDR	AE	MDR	AE	MDR	AE
0.01 m	5.69%	−0.55%	17.82%	0.49%	22.16%	0.25%	13.4%	−1.89%
0.02 m	17.62%	−1.69%	52.18%	−0.41%	54.65%	−0.93%	34.88%	−5.13%
0.03 m	48.09%	−6.07%	103.59%	−15.9%	99.88%	−9.64%	71.98%	−13.54%
0.04 m	97.34%	−25.05%	171.09%	−59.92%	162.98%	−35.86%	124.19%	−29.33%
0.05 m	164.05%	−74.97%	254.58%	−119.4%	243.39%	−81.14%	193.9%	−57.25%

## Data Availability

The raw data supporting the conclusions of this article will be made available by the authors on request.

## Abbreviations

The following abbreviations are used in this manuscript.

ID	Inverse dynamics
RRA	Residual reduction algorithm
GRF	Ground reaction force
IK	Inverse kinematics
STO	Static optimization
MDR	Maximum difference ratio
AE	Assist efficiency
MVC	Maximum voluntary isometric contraction
